# Autologous Fractionated Adipose Tissue as a Natural Biomaterial and Novel One-Step Stem Cell Therapy for Repairing Articular Cartilage Defects

**DOI:** 10.3389/fcell.2020.00694

**Published:** 2020-07-31

**Authors:** Qi Li, Fengyuan Zhao, Zong Li, Xiaoning Duan, Jin Cheng, Jiahao Zhang, Xin Fu, Jiying Zhang, Zhenxing Shao, Qinwei Guo, Xiaoqing Hu, Yingfang Ao

**Affiliations:** Institute of Sports Medicine, Beijing Key Laboratory of Sports Injuries, Peking University Third Hospital, Beijing, China

**Keywords:** adipose tissue, stem cell, stromal vascular fraction, cartilage regeneration, natural biomaterial

## Abstract

Articular cartilage damage remains a tough challenge for clinicians. Stem cells have emerged promising biologics in regenerative medicine. Previous research has widely demonstrated that adipose-derived mesenchymal stem cells (ADSCs) can promote cartilage repair due to their multipotency. However, enzymatic isolation and monolayer expansion of ADSCs decrease their differentiation potential and limit their clinical application. Here, a novel adipose tissue-derived product, extracellular matrix/stromal vascular fraction gel (ECM/SVF-gel), was obtained by simple mechanical shifting and centrifugation to separate the fat oil and concentrate the effective constituents. This study aimed to evaluate the therapeutic effect of this natural biomaterial on the repair of articular cartilage defects. Scanning electron microscopy showed that the fibrous structure in the ECM/SVF-gel was preserved. ADSCs sprouted from the ECM/SVF-gel were characterized by their ability of differentiation into chondrocytes, osteoblasts, and adipocytes. In a rabbit model, critical-sized cartilage defects (diameter, 4 mm; depth, 1.5 mm) were created and treated with microfracture (MF) or a combination of autologous ECM/SVF-gel injection. The knee joints were evaluated at 6 and 12 weeks through magnetic resonance imaging, macroscopic observation, histology, and immunohistochemistry. The International Cartilage Repair Society score and histological score were significantly higher in the ECM/SVF-gel group than those in the MF-treated group. The ECM/SVF-gel distinctly improved cartilage regeneration, integration with surrounding normal cartilage, and the expression of hyaline cartilage marker, type II collagen, in comparison with the MF treatment alone. Overall, the ready-to-use ECM/SVF-gel is a promising therapeutic strategy to facilitate articular cartilage regeneration. Moreover, due to the simple, time-sparing, cost-effective, enzyme-free, and minimally invasive preparation process, this gel provides a valuable alternative to stem cell-based therapy for clinical translation.

## Introduction

Articular cartilage defects in the knee joint are a common clinical problem, which can result in severe pain, joint swelling, substantial reduction in mobility, further joint deterioration, and progression towards osteoarthritis (Dai et al., 2014; Kwon et al., 2019). Due to its avascular and aneural nature with low cellularity, articular cartilage is difficult to self-heal. The current clinical and pre-clinical strategies for cartilage tissue regeneration mainly include microfracture (MF), autologous chondrocyte implantation (ACI), stem cell therapy, autologous cartilage chip (ACC), allograft cartilage, and scaffold-based tissue engineering techniques (Makris et al., 2015). However, each treatment has some limitations. The clinical outcomes of MF and ACI in a large number of cases show that the fibrocartilage tissue formed and its biomechanical properties are inferior to those of native articular cartilage (Bianchi et al., 2019). Cell-based therapy including ACI and stem cell implantation consists of two stages: primary cell culture and *ex vivo* expansion for a large quantity, and the implantation procedure. Furthermore, dedifferentiation and senescence during cell expansion, high costs, long wait times, and two-stage operation limit their wide clinical use. ACC and allografts such as the recently reported particulate juvenile allograft cartilage, have been proven to have a good repair effect; however, donor-site complications or tissue source are major limitations (Ao et al., 2019). To date, none of these treatments are able to fully restore injured articular cartilage (Wang X. et al., 2019).

In the past two decades, mesenchymal stem cells (MSCs) have been considered one of the most promising treatments for cartilage injuries due to their self-renewal capability, high plasticity, and immunosuppressive, anti-inflammatory action, and multipotent differentiation ability into selected lineages including chondrocytes (Kondo et al., 2019). First described in 2001, MSCs derived from adipose (ADSCs) were found to be superior candidate due to their easy acquisition and good regenerative effect (Cui et al., 2009). Recently, a cohort study demonstrated comparable clinical outcomes of the stromal vascular fraction (SVF) isolated from adipose tissue without primary culture and further expansion, with ADSCs for the treatment of knee osteoarthritis (Yokota et al., 2019). Since 2006, the US FDA has promulgated regulations to prevent the risk of transmitting contamination or genetic damage from stem cells (Halme and Kessler, 2006). Preparation of both ADSCs and SVF requires the process of exogenous enzymatic digestion, increasing the potential risk of infection and requiring a rigorous approval process before clinical application. Therefore, an enzyme-free method with minimal manipulation for ADSCs or SVF needs to be developed. Actually, adipose tissue is a source of stem cells niche (Berry et al., 2016), and provides a native scaffold consisting of extracellular matrix (ECM) elements that support structural architecture and biological function (Yu et al., 2013). Based on this principle, researchers have developed nanofat from lipoaspirate grafting between two syringes, and micro-fragmented adipose tissue from a commercial device kit. Recently, these have shown attractive potential in regenerative medicine including promotion of wound healing (Yao et al., 2017; Lonardi et al., 2019), improving ischemic flap survival (Zhang et al., 2018), and remolding bone tissue formation (Guerrero et al., 2018). However, it is still unclear whether autologous fractionated adipose tissue can provide new treatment options to repair articular cartilage defects.

In this study, autologous adipose tissue was mechanically processed and centrifuged to form a novel ready-to-use ECM/SVF-gel with a short preparation time without enzymatic digestion, additional cell expansion, or other complex manipulations. The ECM/SVF-gel could provide a three-dimensional ECM environment as well as intrinsic ADSCs for the regeneration of hyaline-like cartilage. To the best of our knowledge, this is the first attempt to investigate autologous fractionated adipose tissue with a novel and simple enzyme-free technique for cartilage tissue engineering. We hypothesized that the ECM/SVF-gel would promote cartilage repair compared to the conventional MF treatment alone.

## Materials and Methods

### Animals

This study was approved by the Peking University Biomedical Ethics Committee. All animals were purchased from Peking University Animal Administration Center and all procedures were performed according to the guidelines for the Care and Use of Laboratory Animals (National Academies Press, National Institutes of Health Publication No. 85-23, revised 1996). Adult male New Zealand white rabbits weighing 2.7–3.2 kg (5–6 months) were housed individually with free access to diet and activities.

### ECM/SVF-Gel Preparation

The ECM/SVF-gel was prepared as described previously, with some modifications (Yao et al., 2017). Briefly, rabbits were anesthetized by isoflurane inhalation. Their inguinal area was shaved and prepared for aseptic surgery. In total, approximately 5 mL of inguinal adipose tissue was collected and finely minced using ophthalmic scissors. Minced fat tissue was then transferred to two10-mL syringes connected by a Luer-Lock connector with an internal diameter of 2 mm. After mechanical shifting for 90 times, the emulsified fat was filtered to remove the connective tissue remnants and was centrifuged at 2000 × *g* for 3 min. The sticky mixture below the oil layer was defined as the ECM/SVF-gel and was collected for further use.

### Rheological Test

To verify the physical properties of the ECM/SVF-gel, rheological test was carried out on a rheometer (HAAKE MARS III, Thermo Fisher Scientific, Karlsruhe, Germany). The storage modulus G′ and loss modulus G″ of the ECM/SVF-gel were measured through frequency sweep analysis. The frequency range was set from 0.1 to 10 Hz at 20 Pa. Three batches of samples were performed with an average of triplicate measurements in each data point.

### Cartilage Defect Model

The cartilage defect model was established as previously described (Shi et al., 2017). The left or right knee joints of thirty rabbits were randomly treated with MF (MF group) or combined with autologous ECM/SVF-gel injection (ECM/SVF-gel group). The MF group served as the control group because MF currently is the most commonly used treatment strategy for cartilage injury (Man et al., 2016). For the ECM/SVF-gel group, the autologous ECM/SVF-gel was prepared as described above. Then, an incision was made on the knee from the lateral side under aseptic conditions and the joint was exposed after the patella was dislocated. Then, a cylindrical defect (4-mm diameter, 1.5-mm depth) was created on the trochlear groove of the distal femur using corneal trephine. Afterwards, standard MF treatment or a combination of 0.1 mL autologous ECM/SVF-gel injection was performed at the defect site. Three MF holes (diameter 0.8 mm, depth 2 mm) were performed and evenly distributed within the defect. Finally, the joint was closed with a suture; penicillin was administered intramuscularly for 3 days to avoid infection. All rabbits were kept in their individual cages with free access to food and water before they were sacrificed at 6 weeks or 12 weeks post-operation.

### Cell Culture and Multilineage Differentiation

The ECM/SVF-gel was allowed to attach to 25 cm^2^ culture flasks containing Minimum Essential Medium-alpha (Gibco, Grand Island, NY, United States) supplemented with 10% fetal bovine serum and 1% penicillin/streptomycin (100 units/ml penicillin and 100 μg/ml streptomycin). The culture flasks were maintained at 37°C in a humidified incubator containing 5% CO_2_ with the culture medium changed every 3 days. When the cells reached 90% confluence, they were harvested for multi-lineage differentiation assays including adipogenesis, osteogenesis, and chondrogenesis as described previously (Hu et al., 2015). Briefly, ADSCs were seeded in a 6-well plate at a density of 1.0 × 10^5^ cells/well with adipogenic or osteogenic differentiation medium (Cyagen Biosciences, Guangzhou, China). After three weeks of culture, Oil red O staining and Alizarin red staining were performed to assess adipogenesis and osteogenesis, respectively. For chondrogenesis, micromass culture was performed. Cell suspension droplets (5 μL, with 1.0 × 10^7^ cells/mL) were pipetted in the center of a 24-well plate. After allowing to attach for 4 h, chondrogenic differentiation medium (Cyagen Biosciences) was carefully added and changed once every 3 days. Alcian blue staining was then performed to assess the glycosaminoglycan formation after a 21-day chondrogenic induction.

### Flow Cytometry

Cell surface antigen markers was detected by flow cytometry. Briefly, cells were harvested with 0.25% trypsin, centrifuged at 300 × *g* for 5 min, and then incubated with CD29 (Milliopre, MAB1951F, Bedford, MA, United States), CD90 (Abcam, ab226, Cambridge, United Kingdom), CD105 (GeneTex, GTX11415, Irvine, CA, United States), CD34 (eBioscience, MA1-22646, Carlsbad, CA, United States), and CD45 (eBioscience, MHCD4501) for 1 h, respectively. Fluorescence was detected by a FACSVerse flow cytometer (BD Biosciences, San Jose, CA, United States). Data acquisition and analysis were performed using the FlowJo software (Tree Star, Ashland, OR, United States) (Li Q. et al., 2019).

### Scanning Electron Microscopy (SEM)

Morphological characteristics of the ECM/SVF-gel were examined using SEM. Briefly, specimens were fixed with 4% glutaraldehyde and post-fixed in 1% osmium tetroxide for 2 h at 4°C. After dehydration in a gradient series of ethanol, samples were sputter-coated with a 5-nm layer of gold in a high-vacuum gold sputter coater, and were then examined using a JSM-7900F scanning electron microscope (JEOL, Tokyo, Japan).

### Magnetic Resonance Imaging (MRI)

At 6 and 12 weeks post-surgery, knee samples from each group underwent MRI analysis. All examinations were performed with a Siemens TIM Trio 3.0 T (T) MRI scanner (Siemens, Erlangen, Germany) using a small animal-specific coil. Morphological characteristics of neo-cartilage were evaluated under optimized imaging parameters (Supplementary Table S1) as described previously (Huang et al., 2014).

### Gross Observation

The distal portion of the femurs in each group was carefully dissected and photographed after MRI scanning. The gross morphology was blindly evaluated by 2 observers. Semiquantitative analysis was then performed based on the degree of defect repair, integration with border zone, and macroscopic appearance (Supplementary Table S2) according to the International Cartilage Repair Society (ICRS) scoring system (van den Borne et al., 2007).

### Histological Evaluation

At different time points, the repaired knees (*n* = 5 in each group) were harvested and fixed in 10% neutral buffered formalin for 48 h. Subsequently, whole specimens were demineralized in a decalcifying solution (ZSGB-BIO, Beijing, China) for two weeks. The decalcified specimens were then trimmed, dehydrated in a graded ethanol series, and embedded in paraffin. Serial sections of 5-μm thickness were cut and stained with hematoxylin and eosin (H&E) and toluidine blue (TB). Immunohistochemistry analysis was performed using antibodies against type II collagen (Invitrogen, MA5-13026, Carlsbad, CA, United States), type I collagen (Sigma-Aldrich, C2456, St. Louis, MO, United States), and type X collagen (Abcam, ab49945). A modified scoring system (Supplementary Table S3) was used to assess the histological repair outcomes of articular cartilage defects (Wakitani et al., 1994). Histological evaluation was performed by the same 2 observers in a blinded manner.

### Nanoindentation Assessment of Repaired Cartilage

Biomechanical properties of regenerated tissues were evaluated using nanoindentation tests at 12 weeks (*n* = 5 in each group) post-operatively as described in previous reports (Man et al., 2016; Meng et al., 2017). The samples were isolated from the central area of repaired tissues and normal cartilage, and were kept hydrated with phosphate buffered saline solution at room temperature. The specimens were then examined using the TI 950 TriboIndenter In-Situ Nanomechanical Test System (Hysitron Inc., Minneapolis, MN, United States) using a conospherical diamond probe tip. Each indentation was force-controlled to a maximum indentation depth of 500 nm. The load procedure was applied to each specimen with loading (5 s), hold (2 s), and unloading (5 s).

### Statistical Analysis

Data are expressed as the mean ± standard deviation (SD). Analyses were performed using the SPSS 22.0 software. Statistical significance (*P* < 0.05) was calculated using unpaired two-tailed Student’s *t*-tests (two groups) or one-way ANOVA (homogeneity of variance, three groups).

## Results

### Preparation and Characterization of ECM/SVF-gel

**FIGURE 1:**
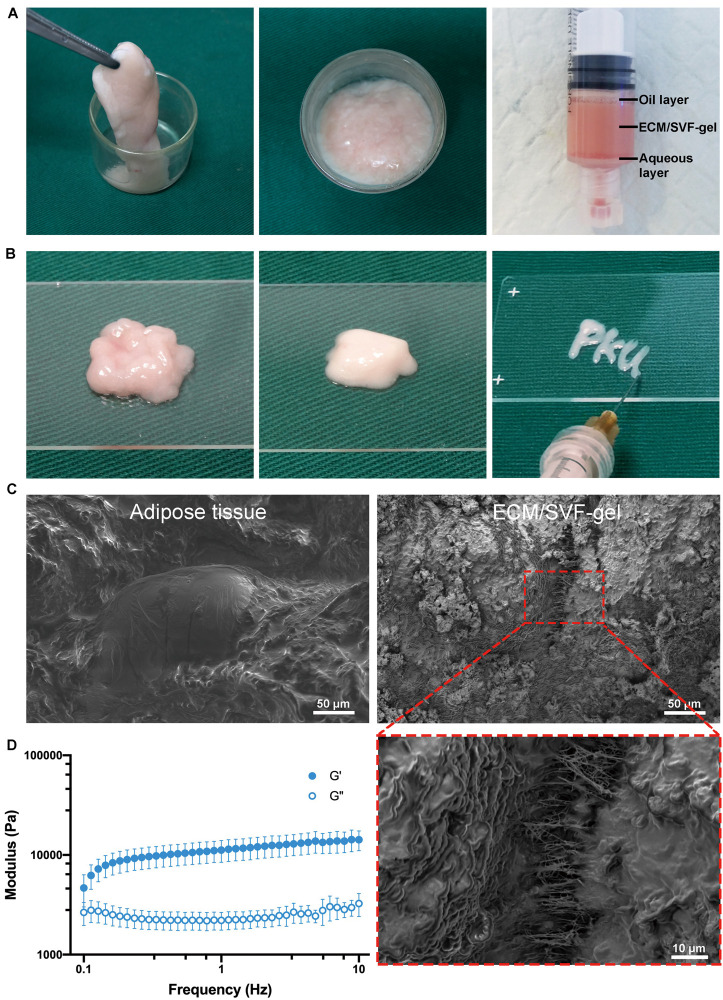
Preparation and morphologic characteristics of the ECM/SVF-gel. **(A)** Rabbit adipose tissue was sufficiently minced. After shifting and centrifugation, the ECM/SVF-gel was in the middle layer. **(B)** Micrographic appearance of minced fat and ECM/SVF-gel. The ECM/SVF-gel could be injected easily through a 27G needle. **(C)** Scanning electron microscopy showed that minced adipose tissue maintained an intact structure, whereas the ECM/SVF-gel had loose and porous extracellular fibers. **(D)** Rheological test for the ECM/SVF-gel (*n* = 3).

After mincing, shifting and centrifugation, the adipose tissue was divided into three layers: the upper oil phase, the middle ECM/SVF-gel, and the lower aqueous phase (Figure 1A). Compared to the minced adipose tissue, ECM/SVF-gel formed a jellylike gel with a smooth texture. The ECM/SVF-gel was transferred to a 1-mL syringe and could be easily injected the letters “PKU” shape through a 27-gauge needle (Figure 1B and Supplementary Video), indicating a good injectable property in the final product. Scanning electron microscopy was conducted to observe alterations in the ECM structure after the mechanical shifting process. The minced adipose showed an intact structure, whereas the ECM/SVF-gel had loose and porous extracellular fibers (Figure 1C). Gel usually has a typical characteristic that the storage modulus G′ is higher than the loss modulus G″, while the solution has the opposite property. Then, we performed rheological test to assess the physical properties of the ECM/SVF-gel. Our data showed that the storage modulus G′ was higher compared with the loss modulus G″ in the ECM/SVF-gel (Figure 1D), indicating its gel property.

### Multilineage Differentiation Potential and Surface Marker of ECM/SVF-Gel Derived ADSCs

**FIGURE 2:**
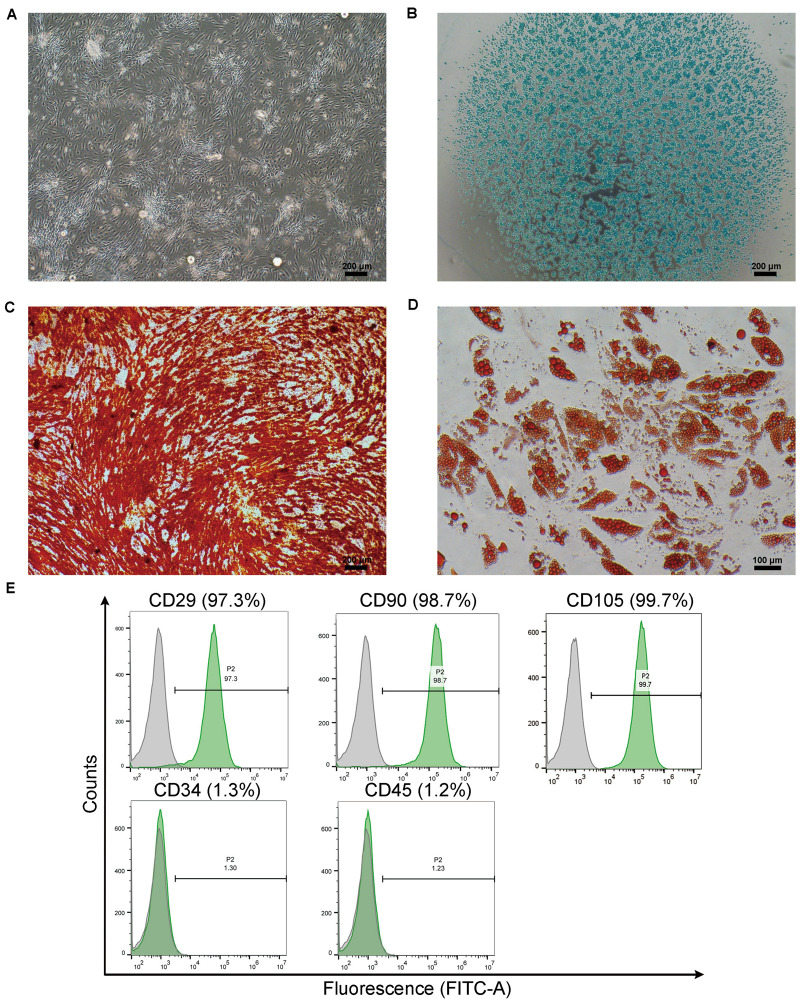
Multi-lineage differentiation of cultured ADSCs migrated from the ECM/SVF-gel. **(A)** Cultured primary ADSCs from the ECM/SVF-gel exhibited a typical spindle shape morphology. **(B)** Chondrogenic, **(C)** osteogenic, and **(D)** adipogenic differentiation of ADSCs was induced and determined by Alcian blue staining for glycosaminoglycans, Alizarin red staining for matrix mineralization, and Oil Red O staining for intracellular lipid droplets, respectively. Scale bars: **(A–C)** = 200 μm; **(D)** = 100 μm. **(E)** Flow cytometry analysis of surface marker on ADSCs from the ECM/SVF-gel.

The ADSCs isolated from ECM/SVF-gel were cultured for 7–14 days and representative cells were photographed by light microscopy. These cells showed a typical spindle-shaped morphology (Figure 2A). To verify whether these ADSCs possessed of multipotential differentiation capability, cells were, respectively, incubated in chondrogenic, osteogenic, and adipogenic medium. Positive results of Alcian blue, Alizarin red, and Oil Red O staining demonstrated that ADSCs successfully differentiated into chondrocytes (Figure 2B), osteocytes (Figure 2C), and adipocytes (Figure 2D), respectively. The results of flow cytometry analysis demonstrated that MSCs positive phenotypic markers of CD29 (97.3%), CD90 (98.7%) and CD105 (99.7%) were overexpressed, whereas the hematopoietic antigen CD34 (1.3%) and the leukocyte common antigen CD45 (1.2%) were negatively expressed (Figure 2E).

### MRI Observations of Repaired Knees

**FIGURE 3:**
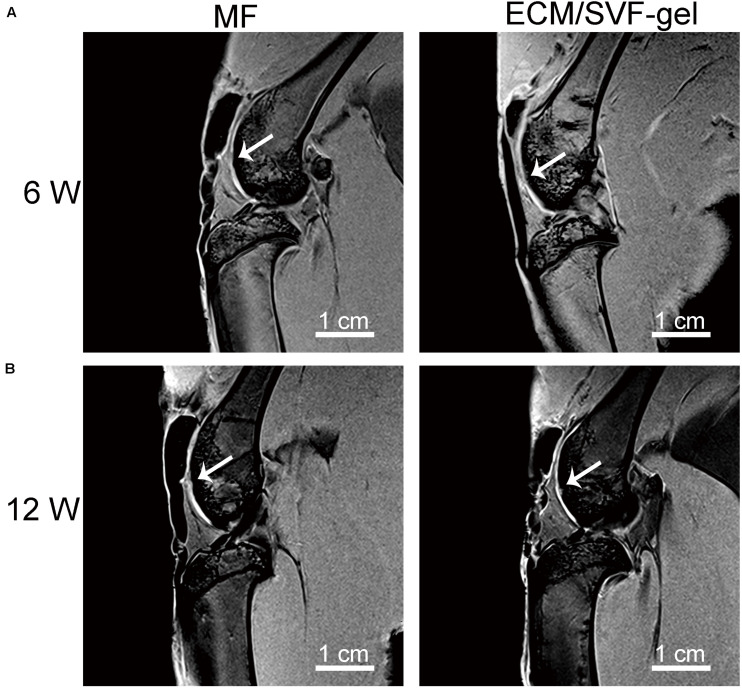
High-resolution MRI of repaired knees. Representative MRI images at 6 weeks **(A)** and 12 weeks **(B)** post-operation. White arrow, repaired sites of articular cartilage; scale bars = 1 cm.

As shown in Figure 3A, high-resolution MRI images demonstrated that the defects in the MF group were poorly filled at 6 weeks postoperatively, whereas those in the ECM/SVF-gel group were almost completely filled with a smooth surface, though not up to the joint surface. At 12 weeks, the injuries in the MF group were irregularly filled with a rough surface. In contrast, uniform and complete filling of cartilage repair was observed in the ECM/SVF-gel group. Furthermore, the signal intensity of the repaired tissue in the ECM/SVF-gel group was similar to that of the adjacent normal cartilage (Figure 3B). Collectively, the MRI results indicate that the ECM/SVF-gel had a better effect of cartilage repair compared to the traditional MF treatment that is widely used in the clinic.

### Gross Evaluation of Cartilage Repair

**FIGURE 4:**
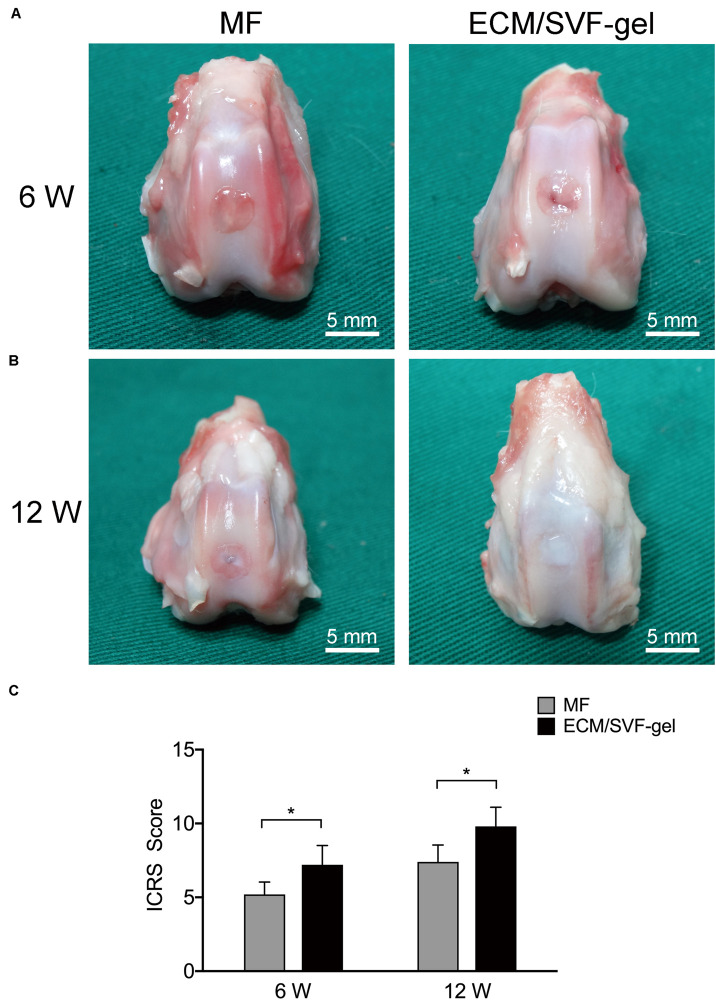
Macroscopic evaluation and ICRS score. **(A)** At 6 weeks and **(B)** 12 weeks, repaired knees were observed and photographed. Scale bars = 5 mm. **(C)** ICRS gross scoring of cartilage repair. Results are presented as the mean ± SD; *n* = 5; *p < 0.05.

No complications were observed in any of the animals. At 6 weeks after surgery, the defects in the MF group were filled with a few blood clot-like tissues, whereas the filling in the ECM/SVF-gel group was more complete and uniform. The boundaries of the repaired sites were still obvious in both groups at 6 weeks (Figure 4A). At 12 weeks, the regenerated tissue in the MF group showed a rough surface and recognizable margin. However, in the ECM/SVF-gel group, newly regenerated tissue with a smooth surface showed integration with the surrounding area and had a color and texture similar to normal cartilage, indicating the formation of hyaline cartilage-like tissue (Figure 4B and Supplementary Figure S1). These findings suggest that the ECM/SVF-gel may effectively promote the filling of cartilage defects and facilitate neo-cartilage regeneration.

The ICRS scoring results were consistent with the macroscopic evaluations. As shown in Figure 4C, ICRS scores of the 6-week repaired tissues in the ECM/SVF-gel group (7.20 ± 1.3) were significant higher (*P* < 0.05) than those in the MF group (5.2 ± 0.8). The difference in the scores between the MF group (7.4 ± 1.1) and the ECM/SVF-gel group (9.8 ± 1.3) was significant (*P* < 0.05) at 12 weeks post-surgery, demonstrating that the repaired tissues of the ECM/SVF-gel group were considered as nearly normal (grade II) according to the ICRS overall assessment (Supplementary Table S2).

### Histological Assessment of Cartilage Repair

**FIGURE 5:**
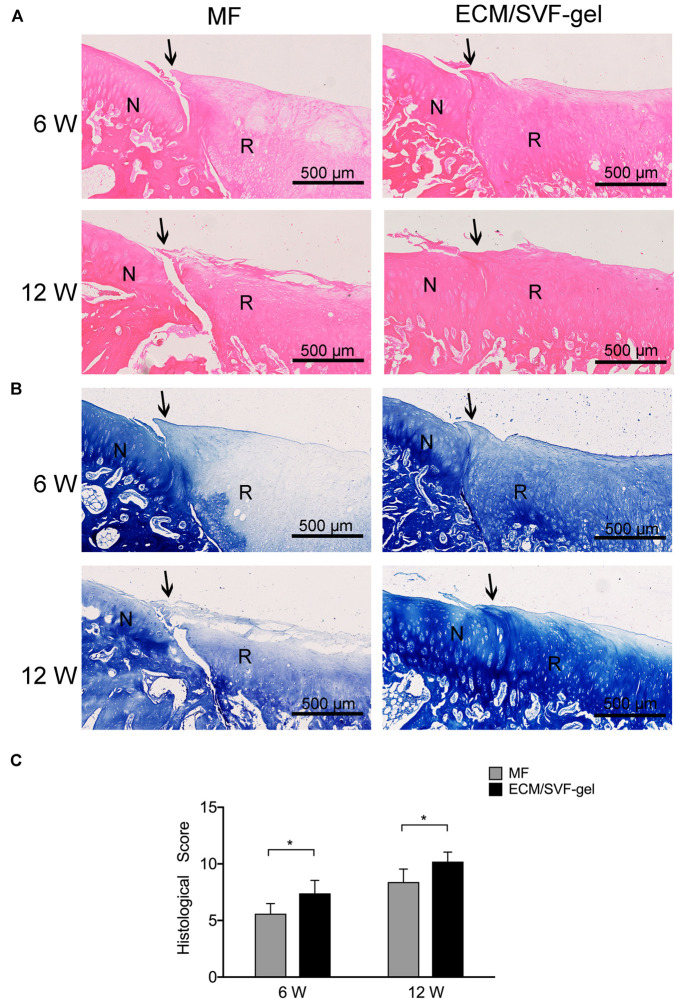
Histological assessment of repaired cartilage. **(A)** Hematoxylin and eosin staining. N, normal cartilage; R, repair cartilage; the arrows indicate the margins of the normal cartilage and repaired cartilage. **(B)** Toluidine blue staining. **(C)** Histological scores for repaired knees. Data are presented as the mean ± SD; *n* = 5; *P < 0.05.

Hematoxylin and eosin (H&E) staining was performed to evaluate the general repair effects between the two groups (Figure 5A). At 6 weeks post-surgery, the defect in the MF group was poorly filled, with fibrous tissue and a distinct boundary between the normal cartilage and regenerated tissue. However, the defect in the ECM/SVF-gel group was generally filled, and the margin between the normal cartilage and repaired tissue was unclear. At 12 weeks post-surgery, the defect in the MF group was filled with disordered fibrous tissue, and the surface of the repaired tissue was lower than that of the adjacent normal cartilage, which showed a degenerative architecture. In contrast, in the ECM/SVF-gel group, the reparative tissue was more similar to the surrounding host cartilage, and highly organized chondrocyte-like cells were observed (Supplementary Figure S2).

Further, toluidine blue staining was performed to assess the content of glycosaminoglycan, an important component of the cartilage matrix (Figure 5B). In the MF group, weak staining was observed at 6 weeks and 12 weeks post-operation, which indicated poor glycosaminoglycan deposition. After 12 weeks of surgery, the regenerated tissue in the ECM/SVF-gel group displayed strong positive and uniform staining, which was similar to the surrounding normal cartilage (Supplementary Figure S3).

A modified scoring system was used to measure the histological outcomes of cartilage repair (Figure 5C). Consistent with the results shown above, the ECM/SVF-gel group had significantly higher (*P* < 0.05) histological scores compared with the MF group at 6 weeks (7.4 ± 1.1 *vs.* 5.6 ± 0.9) and 12 weeks (10.2 ± 0.8 *vs.* 8.4 ± 1.1).

**FIGURE 6:**
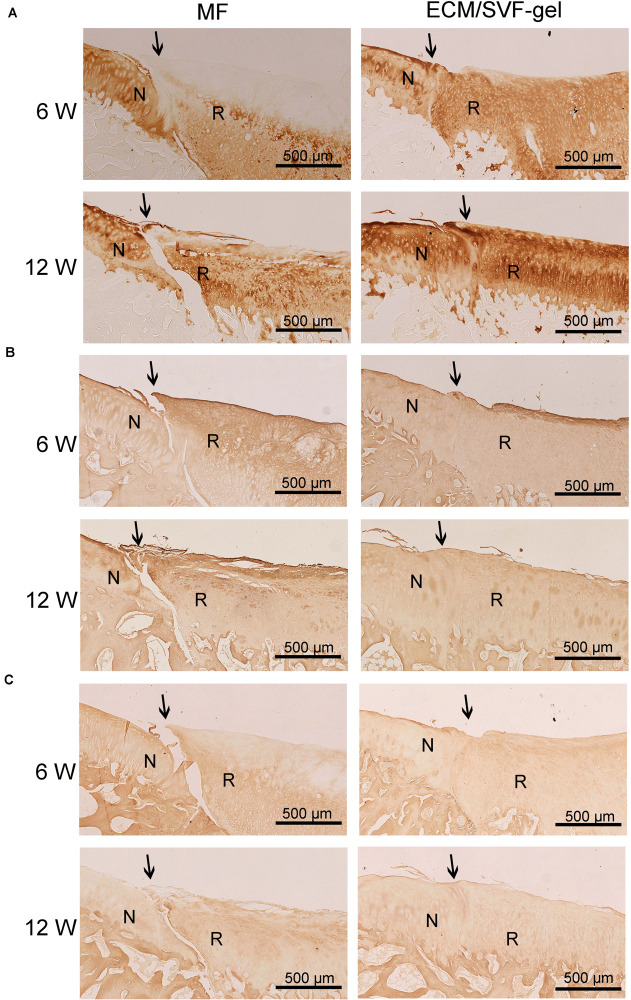
Immunohistochemistry for regenerated tissue. **(A)** COL II, **(B)** COL I, and **(C)** COL X of specimens in each group were detected at 6 and 12 weeks after surgery. N, normal cartilage; R, repair cartilage; the arrows indicate the margins of the normal cartilage and repaired cartilage.

Cartilage-specific type II collagen (COL II) was detected by immunohistochemistry to assess the quality of cartilage repair. As shown in Figure 6A and Supplementary Figure S4, COL II expression was observed in the regenerated tissue of both the MF and ECM/SVF-gel groups. At both 6 and 12 weeks after surgery, COL II expression from cells in the ECM/SVF-gel group was higher than that in the MF group, which confirmed the results of H&E and toluidine blue staining. Immunohistochemistry for COL I (Figure 6B and Supplementary Figure S5) was also performed to evaluate the extent of fibrocartilage in the regenerated tissue. In the MF group, the expression of COL I was predominated, suggesting that the main generated component was fibrocartilage. However, the expression of type I collagen was not significant in the ECM/SVF-gel group, indicating the formation of hyaline-like cartilage. Hypertrophy of chondrocytes could disturb articular cartilage regeneration. We further detected hypertrophic marker COL X by immunohistochemistry (Figure 6C and Supplementary Figure S6). The results showed that COL X was slightly stained in both group, and remained no obvious difference compared with the surrounding normal cartilage. Taken together, these findings demonstrated that the ECM/SVF-gel had a better reparative effect on cartilage defects, and that the newly regenerated tissue is closer to normal cartilage.

### Biomechanical Properties of Repaired Cartilage

**FIGURE 7:**
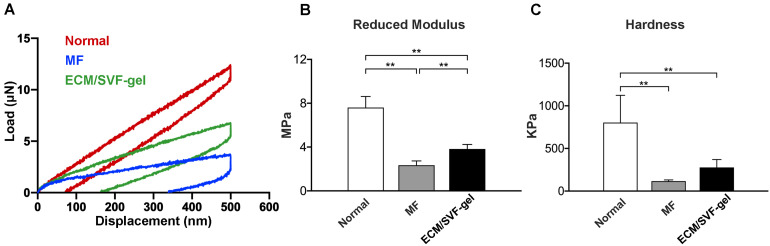
Biomechanical properties of repaired cartilage at 12 weeks post-surgery. **(A)** Typical load-displacement curves obtained with the repaired tissues were compared with those from native cartilage. **(B)** Reduced modulus and **(C)** hardness in each group. Data are presented as the mean ± SD; *n* = 5; **P < 0.01.

At 12 weeks post-surgery, nanoindentation was performed to evaluate the biomechanical properties of the reparative tissue. According to the load-displacement curves (Figure 7A), reduced modulus and hardness were calculated. Normal cartilage displayed the highest reduced modulus, followed by the ECM/SVF-gel group and the lowest in the MF group (Figure 7B). A similar result can be seen in the assessment of hardness (Figure 7C), although there is no statistical difference between the MF and ECM/SVF-gel groups. These data indicate that the ECM/SVF-gel facilitates better biomechanical properties in the repaired tissue.

## Discussion

This study investigated a novel strategy of ready-to-use autologous fractionated adipose tissue named ECM/SVF-gel, and its therapeutic potential as a scaffolding material and novel stem cell-based approach for articular cartilage restoration.

Due to its poor intrinsic healing capacity, damage to the articular cartilage is difficult to cure fully and may induce osteoarthritis, which is predicted to affect more than 67 million people by 2030, leading to costs of more than $3 billion annually in the United States (Madry et al., 2019). There is thus an urgent need to explore new treatment options. In the present study, mechanical grafting was used to disrupt adipose tissue, and centrifugation was performed to remove the released oil and to further increase the density of ECM fibers and stem cells. SEM demonstrated successful concentration of ECM fibers in the ECM/SVF-gel, which could thus provide a scaffolding biomaterial with the microenvironment required for MSCs renewal and differentiation. Consistent with previous studies, we found that ADSCs isolated from ECM/SVF-gel could proliferate and differentiate into various lineages (Zhang et al., 2018; Wang J. et al., 2019), demonstrating that the ECM/SVF-gel preserved the stem cells within adipose tissue. Therefore, the ECM/SVF-gel has attractive prospects as a natural scaffolding biomaterial and stem cell carrier to promote cartilage regeneration. When it comes to the naming of ECM/SVF-gel, we are based on the following reasons. First, the ECM fibers are concentrated and verified by SEM. Second, this method is an enzyme-free approach to isolated SVF cells, which are already demonstrated by some studies (Sun et al., 2017; Yao et al., 2017), and ADSCs isolated in this study are a subset of SVF. Third, gel property is demonstrated by rheological data that the storage modulus G′ was higher than the loss modulus G″. Finally, similar nomenclature has also been reported in other studies (Feng et al., 2019; Wang J. et al., 2019).

To identify the effect on cartilage repair *in vivo*, we injected autologous ECM/SVF-gel into a rabbit cartilage defect model. According to the imaging studies, macroscopic observation and histological examination, the ECM/SVF-gel suggested a superior hyaline-like neo-cartilage restoration compared with the MF treatment alone. Cell migration from adjacent donor cartilage is a key factor driving the process of integration (Pabbruwe et al., 2009). Recently, fragmented adipose tissue was found to significantly accelerated chondrocyte migration into the wound area (Xu et al., 2019), indicating that natural fat derived biomaterials (e.g., ECM/SVF-gel) could recruit chondrocytes to the defect region from the adjacent normal cartilage and promote integration between the repair tissue and surrounding native cartilage. A preclinical study reported that among 130 dogs with spontaneous osteoarthritis, 98% had improved owners’ scores after 6 months with a single injection of fat extract (Zeira et al., 2018). Moreover, there is evidence that fragmented adipose tissue can promote cell proliferation and maintain the stemness of progenitor cells (Randelli et al., 2016). In another study, Desando and colleagues demonstrated that mechanically fragmented adipose tissue-based biomaterials were beneficial for cartilage regeneration due to their trophic activity and the wound-healing activity of CD-163^+^ cells contained in its niche (Desando et al., 2019).

Articular cartilage serves as a cushion to protect the joint from mechanical stresses and strains. Biomechanical performance is a key index for evaluating cartilage tissue engineering (Panadero et al., 2016). In the present work, both reduced modulus and hardness were higher in the ECM/SVF-gel group than those in the MF group; however, they did not reach the levels in normal cartilage. One possible reason is that the observation period of 12 weeks is not long enough to achieve complete healing of the articular cartilage, which needs to be considered in future studies. In addition, although difficult to manage in animal experiments, post-operative rehabilitation is a critical component of the treatment process in the biomechanical recovery of cartilage lesions for patients (Mithoefer et al., 2012).

When it comes to traditional stem cell therapies, cell suspensions show poor adhesion to joints and are easily lost to areas outside the injury site, thereby reducing the effectiveness of treatment. Therefore, it may often be necessary to rely on synthetic bio-scaffolds to promote the cell adhesion or recruitment of seed cells to achieve better repair results (Malda et al., 2019). In ECM/SVF-gels, the preserved natural microenvironment and ADSCs may play a different role when compared with the direct delivery of cell suspensions or combination with other synthetic biomaterials. Meanwhile, during the process of mechanically processing adipose tissues and centrifugation, the final product could be enriched with growth factors and other components such as extracellular vesicles (Xu et al., 2019), which can stimulate tissue regeneration. It is possible that the ECM/SVF-gel facilitates cartilage repair more so through its material characteristics, rather than through its cellular characteristics. Previous studies demonstrated that gels could be used to stabilize the blood clot that forms during microfracture (Hoemann et al., 2007). For example, BST-CarGel (Smith&Nephew) or chitosan gels have been successfully used to stabilize microfracture blood clots in animal models and humans, leading to improved cartilage tissue repair (Stanish et al., 2013; Shive et al., 2015; Méthot et al., 2016; Mohan et al., 2017). The ECM/SVF-gel may stabilize the clot formed by cells that seep from the bone marrow into the defect site, thereby facilitating cartilage repair. Future studies could use cell tracing to determine if the repaired cartilage tissue was derived from cells contained within the ECM/SVF-gel, or if the repair tissue was derived from other cell sources within the host animal. Furthermore, fragmented fat tissue as a natural biomaterial for efficient drug delivery, improved the local drug concentration and prolonged its therapeutic activity (Alessandri et al., 2019), indicating that our ECM/SCF-gel may act as a self-controlled drug release system of intrinsic growth factors to promote cartilage repair. Different from scaffold-based ADSCs treatment (Li X. et al., 2019), this strategy allows overcoming the limitations associated with the enzymatic isolation of SVF cells from adipose tissue and the expansion of ADSCs, as well as the complex process of scaffold fabrication. In addition, a recent study reported that after cryopreservation at −20°C for 3 months without a cryoprotectant, the mechanically processed fat product SVF gel from human lipoaspirate maintained ECM integrity and ADSC viability as well as their multipotency (Feng et al., 2019).

For clinical application, the adipose tissue for ECM/SVF-gel preparation can be sourced from the infrapatellar fat pad of the synovial joint environment or subcutaneous adipose tissue. In clinical practice, during arthroscopic surgery for articular cartilage repair, in order to expose a clear operation field, the infrapatellar fat pad often needs to be removed. Thus, the donor fat is obtained during the debridement process, which is also in line with the fundamental principles of minimally invasive arthroscopic surgery. Moreover, adjacent to the operative knee area, subcutaneous fat around the inner thigh is another choice for surgeons. In addition, liposuction, a technique that has been used for decades in plastic surgery with a very low incidence of major complications, provides an alternative choice of autologous adipose tissue. Some clinicians have recommended the autologous adipose tissue transplantation should be considered for the treatment of delamination and 1st and 2nd degree chondral lesions (Jannelli and Fontana, 2017); however, the exact indication and dose management require additional research in the future.

This study is not without limitations. First, a study with a large animal model and a relatively long observation period beyond 12 weeks needs to be performed in future. As mentioned above, from the perspective of clinical application, the infrapatellar fat pad collected during arthroscopic surgery is the best choice, which also truly meets the one-step procedure. In this study, limited by the small volume of the synovial infrapatellar fat pad in rabbits, we adopted inguinal adipose tissue. Although there may be some differences in the fat from different sites, our strategy was found effective for cartilage repair. In future research, we will consider using larger animals such as pigs for confirming these results. Second, rabbits with fully skeletal maturity should be used in chondral restoration, because the relatively young rabbits used in this study have stronger healing capabilities and may promote better repair results. Third, we did not compare the ECM/SVF-gel with traditional stem cell therapy such as ADSCs injection or SVF transplantation, for cartilage regeneration. Finally, the underlying molecular mechanism of cartilage repair needs to be investigated further.

**FIGURE 8:**
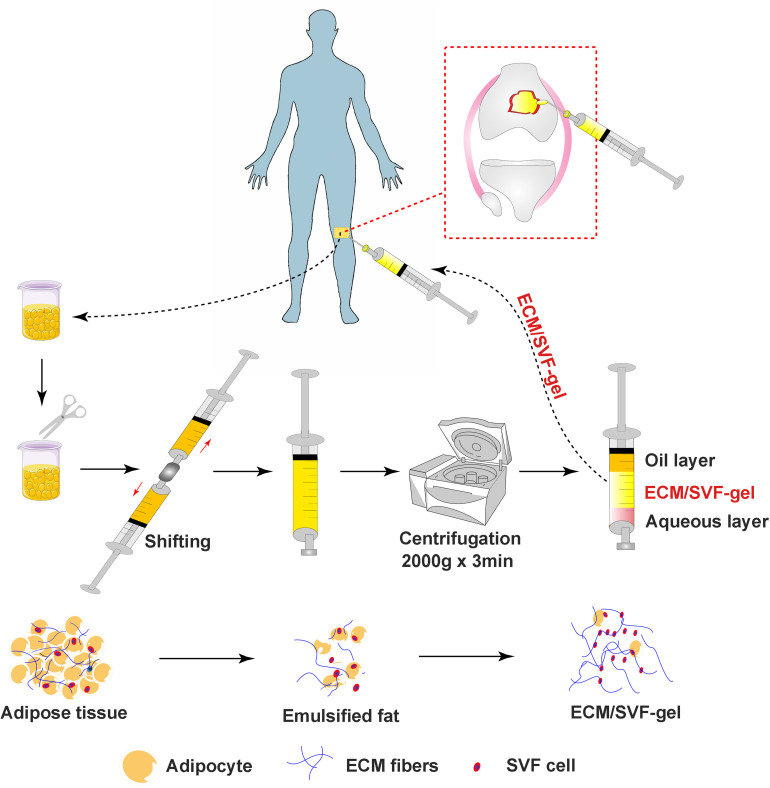
Overview of the clinical application of ECV/SVF-gel for cartilage restoration.

In conclusion, we innovatively propose that application of autologous fractionated adipose tissue (ECM/SVF-gel) could facilitate cartilage injury repair. The ECM/SVF-gel is a minimally manipulated adipose tissue extract that retains the ECM and stem cells after mechanical processing and centrifugation. The results show that the autologous ECM/SVF-gel displays a curative effect on articular cartilage regeneration in a rabbit model. Considering its simple, time-sparing, cost-effective, minimally invasive, and enzyme-free preparation process, this gel may provide a novel concept in the repair of articular cartilage injury (Figure 8). More importantly, the paradigm opens an attractive clinical insight in the potential use of stem cell therapy in regenerative medicine.

## Data Availability Statement

The raw data supporting the conclusions of this article will be made available by the authors, without undue reservation, to any qualified researcher.

## Ethics Statement

The animal study was reviewed and approved by Peking University Biomedical Ethics Committee.

## Author Contributions

YA, XH, and QL conceived the project and designed the experiments. QL, FZ, ZL, XD, JYZ, XF, and JHZ performed the experiments. QL, JC, ZS, and QG collected and analyzed the data. QL wrote the manuscript. YA and XH reviewed the manuscript and supervised the project. All authors contributed to the article and approved the submitted version.

## Conflict of Interest

The authors declare that the research was conducted in the absence of any commercial or financial relationships that could be construed as a potential conflict of interest.
