# Supplementary material for: Autologous Fractionated Adipose Tissue as a Natural Biomaterial and Novel One-Step Stem Cell Therapy for Repairing Articular Cartilage Defects
Source: Front Cell Dev Biol. 2020 Jul 31;8:694. doi: 10.3389/fcell.2020.00694 (PMC7438948; doi:10.3389/fcell.2020.00694)
Supplement: Supplementary file 1 [file Table_1.pdf]

Supplementary Material

Autologous Fractionated Adipose Tissue as a Natural Biomaterial and Novel One-Step Stem Cell Therapy for Repairing Articular Cartilage Defects

Qi Li, Fengyuan Zhao, Zong Li, Xiaoning Duan, Jin Cheng, Jiahao Zhang, Xin Fu, Jiying Zhang, Zhenxing Shao, Qinwei Guo, Xiaoqing Hu* and Yingfang Ao*

Institute of Sports Medicine, Beijing Key Laboratory of Sports Injuries, Peking University Third Hospital, Beijing, China

*** Correspondence:**
Yingfang Ao
aoyingfang@163.com;
and
Xiaoqing Hu
huxiaoqingbd01@sina.com

1. Supplementary Tables

Supplementary Table 1. MRI examination imaging sequences

Series description	Plane	TR (ms)	TE (ms)	FOV (mm)	Flip angle	Slice thickness (mm)	Distance factor (%)	Matrix	BW (Hz/pixel)
Fat-saturated T2-weithted TSE imaging	Sagittal	2000	72	70	150	2	10	256×256	199
PD-weighted TSE imaging	Sagittal	2000	36	70	150	2	10	256×256	199
3D T1-weighted spoiled GRE imaging	Sagittal	26	4.6	60	25	1	20	512×256	200

Abbreviations: TR, time of repetition; TE, time of echo; FOV, field of view; BW, bandwidth; 3D, 3 dimensions; TSE, turbo spin echo; PD, proton density; GRE, gradient echo.

Supplementary Table 2. International Cartilage Repair Society macroscopic evaluation of cartilage repair

Categories	Points
Degree of defect repair	
In level with surrounding cartilage	4
75% repair of defect depth	3
50% repair of defect depth	2
25% repair of defect depth	1
0% repair of defect depth	0
Integration to border zone	
Complete integration with surrounding cartilage	4
Demarcating border < 1 mm	3
3/4 of graft integrated, 1/4 with a notable border > 1 mm width	2
1/2 of graft integrated with surrounding cartilage, 1/2 with a notable border > 1 mm	1
From no contact to 1/4 of graft integrated with surrounding cartilage	0
Macroscopic appearance	
Intact smooth surface	4
Fibrillated surface	3
Small, scattered fissures of cracks	2
Several, small or few but large fissures	1
Total degeneration of grafted area	0
Overall repair assessment	
Grade I: normal	12
Grade II: nearly normal	8-11
Grade III: abnormal	4-7
Grade IV: severely abnormal	1-3

Supplementary Table 3. Histological scoring system of cartilage repair

Categories	Points
Cell morphology	
Hyaline cartilage	4
Mostly hyaline cartilage	3
Mostly fibrocartilage	2
Mostly non-cartilage	1
Non-cartilage only	0
Matrix staining	
Normal	3
Slightly reduced	2
Markedly reduced	1
No metachromatic stain	0
Surface regularity*	
Smooth (>3/4)	3
Moderate (>1/2–3/4)	2
Irregular (1/4–1/2)	1
Severely irregular (<1/4)	0
Thickness of cartilage	
>2/3	2
1/3–2/3	1
<1/3	0
Integration of donor with host adjacent cartilage	
Both edge integrated	2
One edge integrated	1
Neither edge integrated	0
Total maximum	14

*Total smooth area of repair cartilage compared to the entire area of the cartilaginous compartment of the defect.

2. Supplementary Figures

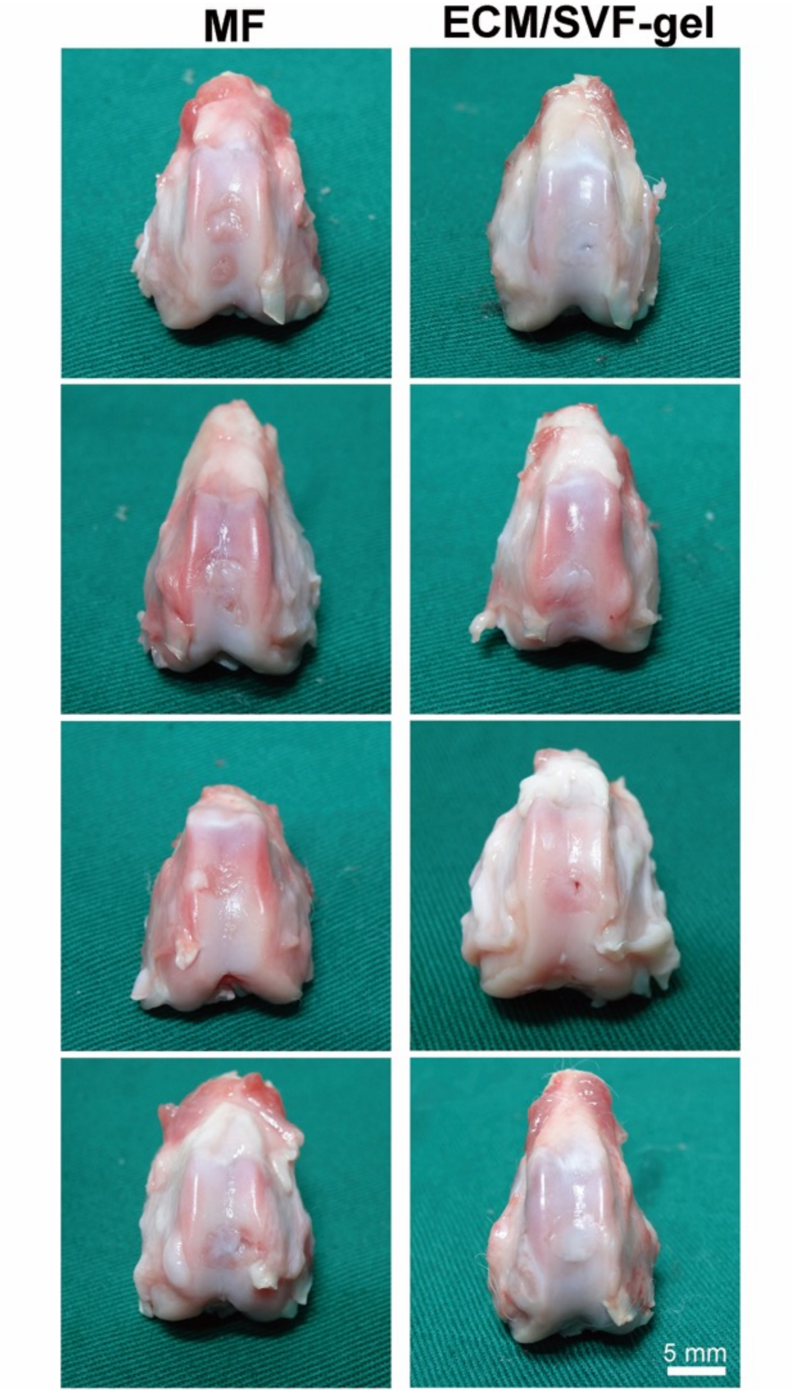

Supplementary Figure 1. Macroscopic photographs for the other donors at 12 weeks after surgery.

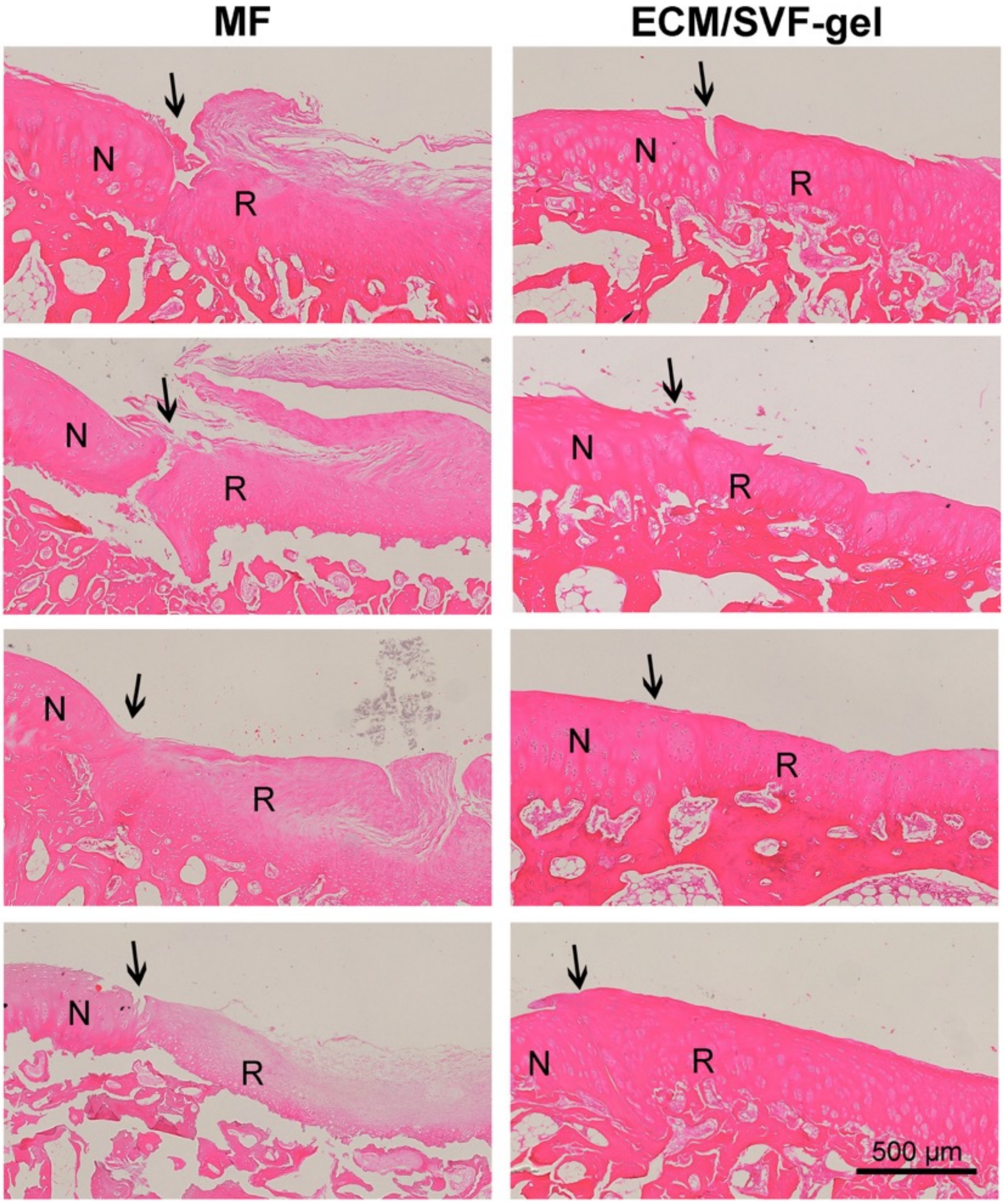

Supplementary Figure 2. Hematoxylin and eosin staining for the other donors at 12 weeks after surgery. N: normal cartilage; R: repair cartilage; the arrows indicate the margins of the normal cartilage and repaired cartilage.

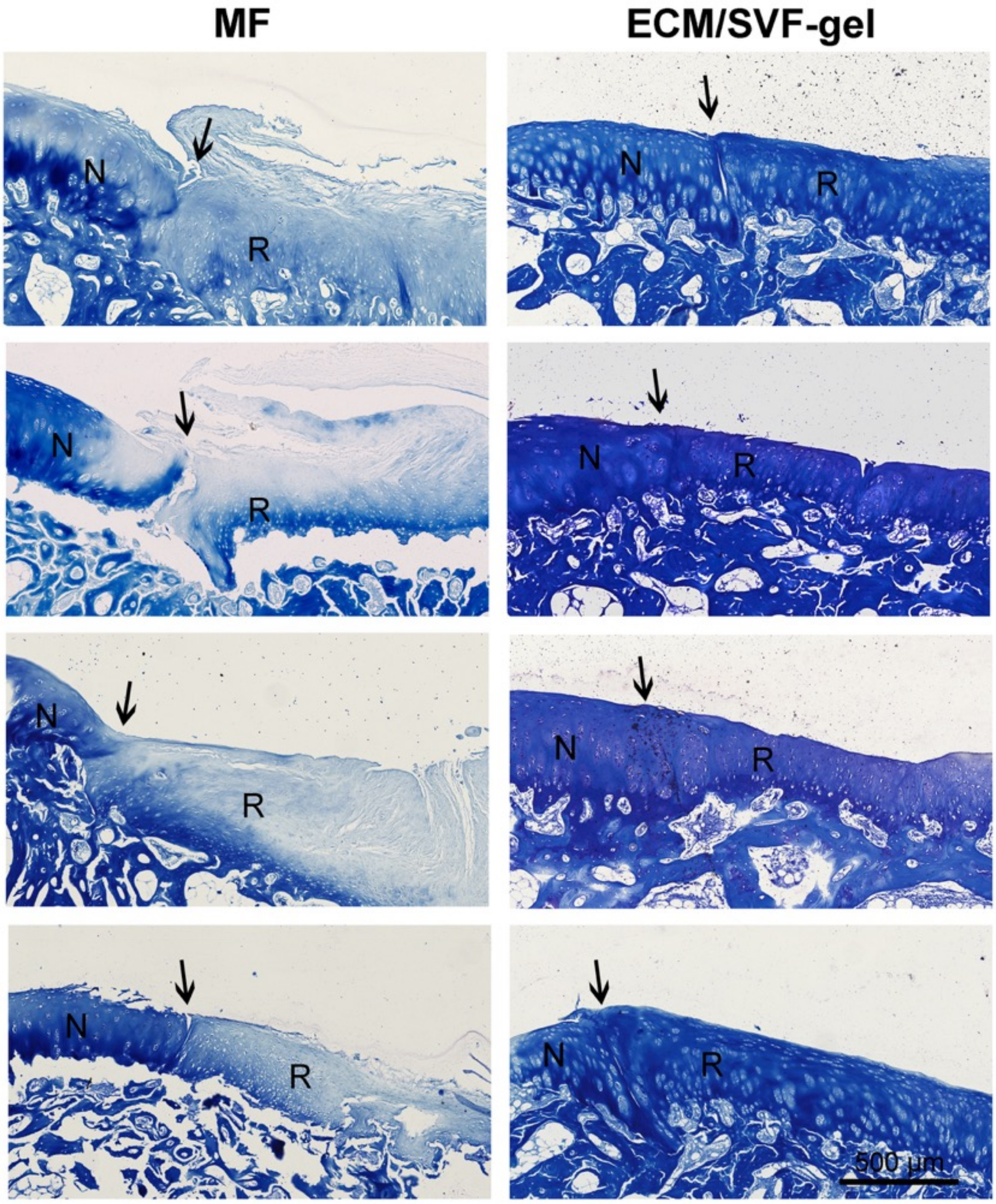

Supplementary Figure 3. Toluidine blue staining for the other donors at 12 weeks post-surgery. N: normal cartilage; R: repair cartilage; the arrows indicate the margins of the normal cartilage and repaired cartilage.

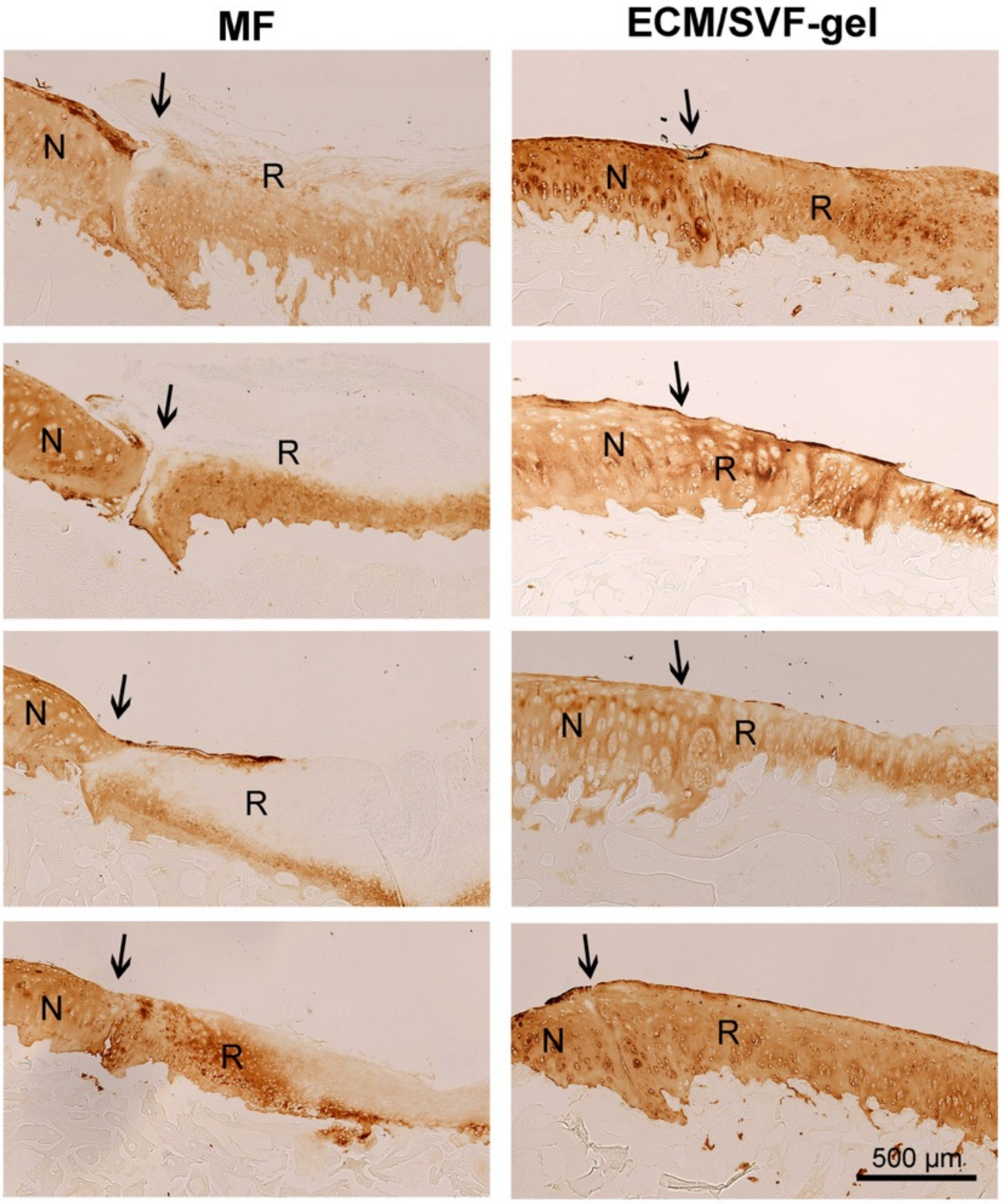

Supplementary Figure 4. Immunohistochemistry staining of COL II for the other donors at 12 weeks after surgery. N: normal cartilage; R: repair cartilage; the arrows indicate the margins of the normal cartilage and repaired cartilage.

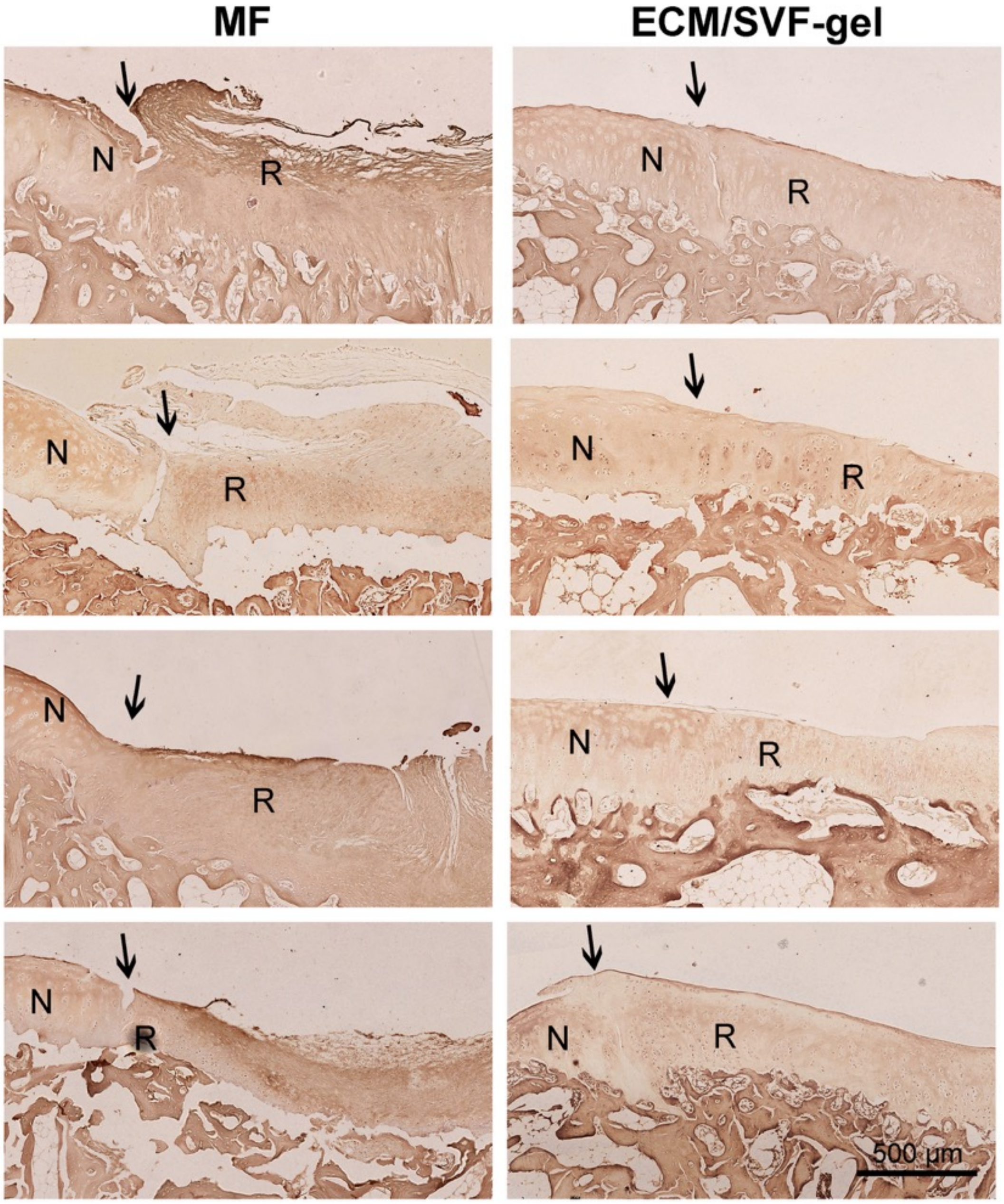

Supplementary Figure 5. Immunohistochemistry staining of COL I for the other donors at 12 weeks post-surgery. N: normal cartilage; R: repair cartilage; the arrows indicate the margins of the normal cartilage and repaired cartilage.

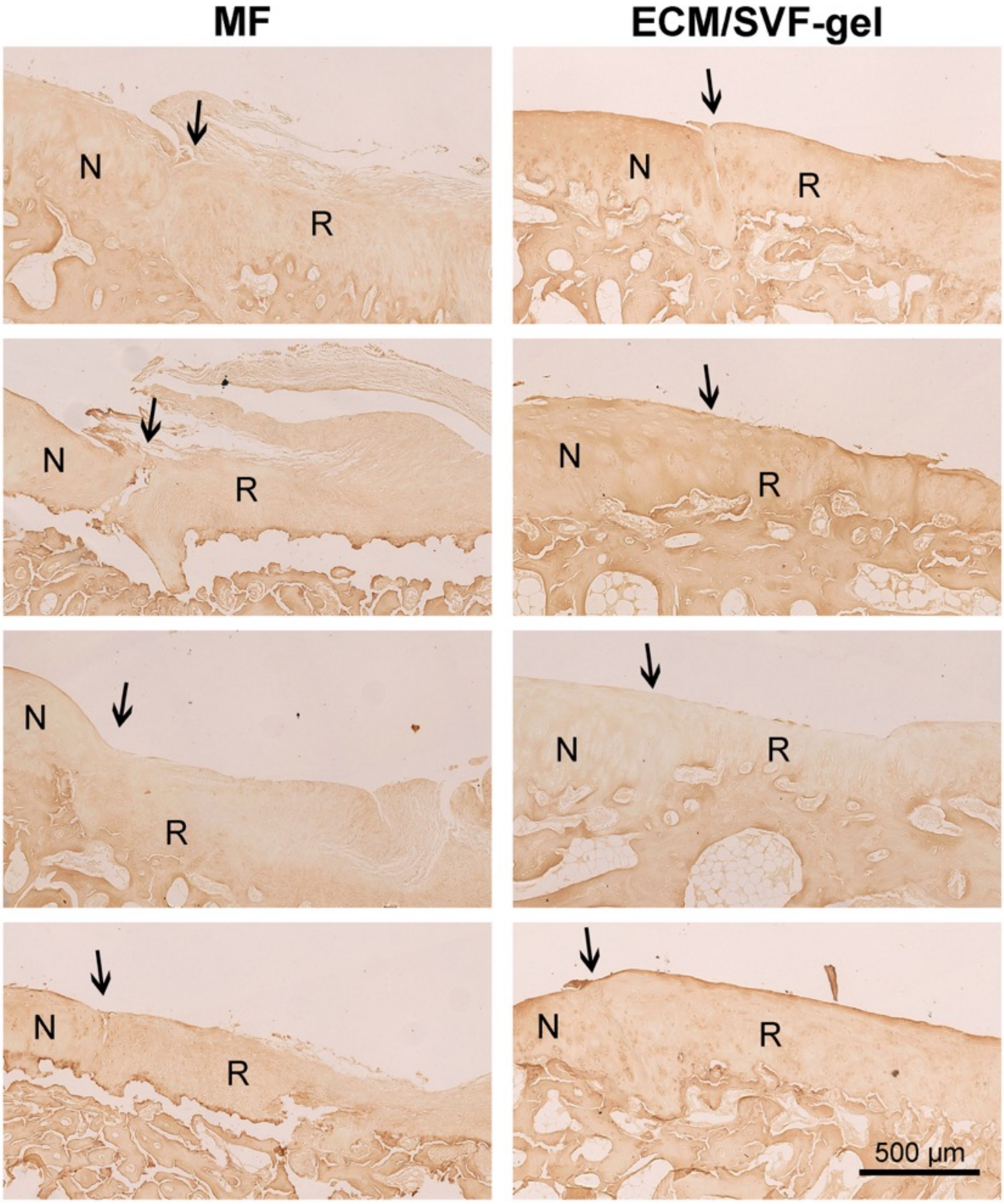

Supplementary Figure 6. Immunohistochemistry staining of COL X for the other donors at 12 weeks after surgery. N: normal cartilage; R: repair cartilage; the arrows indicate the margins of the normal cartilage and repaired cartilage.

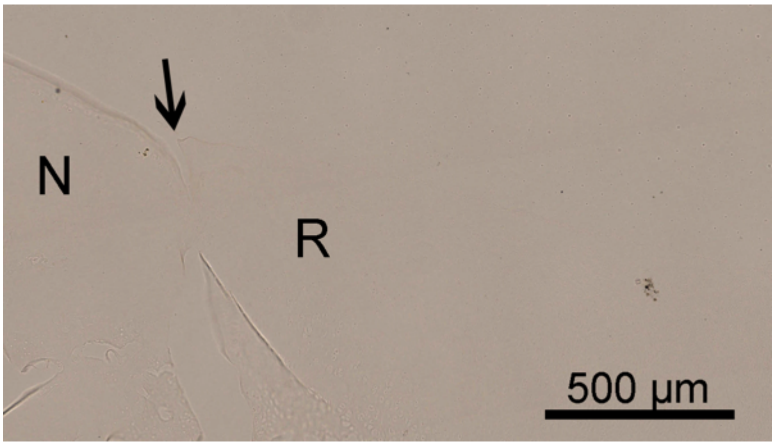

Supplementary Figure 7. Negative control for immunohistochemistry staining. N: normal cartilage; R: repair cartilage; the arrows indicate the margins of the normal cartilage and repaired cartilage.

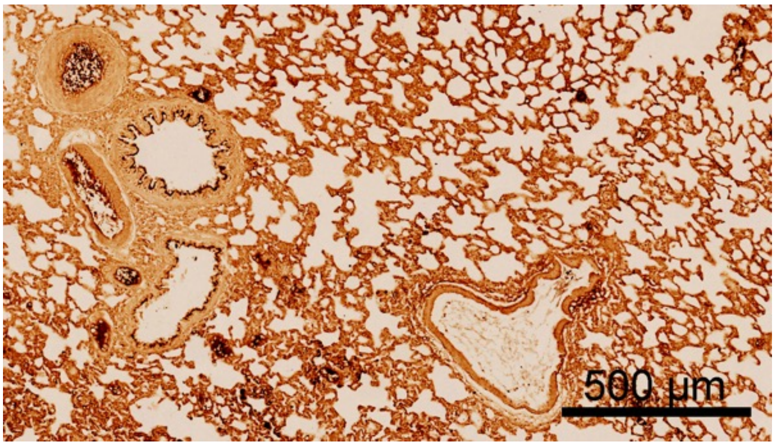

Supplementary Figure 8. Positive control for immunohistochemistry staining of COL X from rat lung tissue.
